# Remarkable Pyro-Catalysis of g-C_3_N_4_ Nanosheets for Dye Decoloration under Room-Temperature Cold–Hot Cycle Excitation

**DOI:** 10.3390/nano13061124

**Published:** 2023-03-21

**Authors:** Zheng Wu, Xiaoyu Shi, Tingting Liu, Xiaoli Xu, Hongjian Yu, Yan Zhang, Laishun Qin, Xiaoping Dong, Yanmin Jia

**Affiliations:** 1 Xi’an Key Laboratory of Textile Chemical Engineering Auxiliaries, School of Environmental and Chemical Engineering, Xi’an Polytechnic University, Xi’an 710048, China; wuzheng@xpu.edu.cn (Z.W.);; 2 College of Materials and Chemistry, China Jiliang University, Hangzhou 310018, China; qinlaishun@cjlu.edu.cn; 3 School of Chemistry and Chemical Engineering, Yangzhou University, Yangzhou 225002, China; yhj@yzu.edu.cn; 4 School of Science, Xi’an University of Posts and Telecommunications, Xi’an 710121, China; 5 Department of Chemistry, School of Sciences, Zhejiang Sci-Tech University, Hangzhou 310018, China; xpdong@zstu.edu.cn

**Keywords:** pyro-catalysis, g-C_3_N_4_ nanosheet, dye decoloration, pyroelectric effect, cold–hot thermal cycle

## Abstract

Pyroelectric materials have the ability to convert the environmental cold–hot thermal energy such as day–night temperature alternation into electrical energy. The novel pyro-catalysis technology can be designed and realized on the basis of the product coupling between pyroelectric and electrochemical redox effects, which is helpful for the actual dye decomposition. The organic two-dimensional (2D) graphic carbon nitride (g-C_3_N_4_), as an analogue of graphite, has attracted considerable interest in the field of material science; however, its pyroelectric effect has rarely been reported. In this work, the remarkable pyro-catalytic performance was achieved in the 2D organic g-C_3_N_4_ nanosheet catalyst materials under the continuous room-temperature cold–hot thermal cycling excitation from 25 °C to 60 °C. The pyro-catalytic RhB dye decoloration efficiency of the 2D organic g-C_3_N_4_ can reach ~92.6%. Active species such as the superoxide radicals and hydroxyl radicals are observed as the intermediate products in the pyro-catalysis process of the 2D organic g-C_3_N_4_ nanosheets. The pyro-catalysis of the 2D organic g-C_3_N_4_ nanosheets provides efficient technology for wastewater treatment applications, utilizing the ambient cold–hot alternation temperature variations in future.

## 1. Introduction

In recent decades, various types of industrial wastewater that contain dyes originating from different sectors such as textile, tanning, printing, food or other industries have resulted in severe and detrimental effects on aquatic life and human ecosystems [1,2,3,4]. Efficient treatment of industrial wastewater has become a topic worth studying due to the negative impact it has on the environment. In order to combat this issue, a variety of physical, chemical and biological treatment methods have been developed and implemented in environmental applications [1,4,5]. The physical method of dye removal, also known as adsorption, involves transferring the dye molecules from the liquid phase to the solid phase using various sorbents such as activated carbon, clay or biomass. However, a major drawback of this method is that it can easily cause secondary pollution due to the disposal or regeneration of the sorbents [6,7]. The chemical method of dye removal consists of two main techniques: photocatalytic decolorization and Fenton oxidation. Photocatalytic decolorization uses light energy to activate a catalyst such as TiO_2_ that can generate reactive oxygen species to degrade dyes. Fenton oxidation uses a mixture of Fe^2+^ and H_2_O_2_ that can produce hydroxyl radicals (•OH) that can also oxidize dyes. Both techniques are non-toxic and effective methods of treating sewage because they can mineralize dyes into harmless substances such as CO_2_ and H_2_O. However, they also face some challenges such as low reaction efficiency due to recombination of carriers, high cost due to catalyst consumption or recovery, and conditional use due to pH or light requirements [8,9,10,11,12]. Biological methods of dye removal involve using microorganisms or enzymes to degrade dyes into simpler and less harmful compounds. However, these methods have some major drawbacks that limit their applicability to most types of dye effluents. One of these drawbacks is that modern dyes are often stable and resistant to biodegradation due to their complex molecular structures and synthetic origins. Another drawback is that most industrial dyes are toxic and inhibitory to the organisms or enzymes used in these processes, affecting their growth and activity. Therefore, biological methods are not suitable for treating wastewater containing high concentrations or diverse mixtures of dyes [5,7,8,9]. In view of the limitations and drawbacks of the existing methods of sewage treatment, especially for dye removal, there is an urgent need to develop new and environmentally friendly methods that can overcome these challenges. Such methods should be able to degrade dyes completely and efficiently without producing harmful by-products or secondary pollution. They should also be cost effective, scalable and adaptable to different types of dyes and wastewater conditions.

Thermal energy is a common and easily accessible source of energy that can be obtained from nature with minimal effort. It can be used to perform green, self-powered catalytic reactions that utilize waste heat from the environment. These reactions are known as pyro-catalysis. However, pyro-catalysis is mainly achieved by pyrolyzing organic materials to break down organic materials into smaller molecules, which usually requires high temperatures to overcome the activation energy barriers of the reactions (400–1000 °C) [10]. To date, reports on pyro-catalysis at room temperature have been extremely limited. If thermal catalysis can be performed at room temperature, the environmental waste heat energy can be collected from a wider range of sources, which is more practical.

Cold–hot alternation such as day–night changes can be also designed to drive chemical catalysis reaction via the recent novel technology of pyro-catalysis, in which the pyroelectric catalyst is used. Pyroelectric materials have a strong ability to convert cold–hot thermal energy into electrical energy [11]. Pyro-catalysis is based on the pyroelectric properties of materials, generating positive and negative charges under the drive of temperature variation. The induced positive and negative electric charges, together with dissolved oxygen and hydroxide ions in the solution, generate strong oxidizing radicals for degrading dye wastewater [11]. In addition, pyroelectric materials have a high energy conversion efficiency of 40–45% between the thermal energy and the electric energy [12], which is far higher than that of photovoltaic materials < 20% [13]. In theory, the pyroelectrically induced pyro-catalytic effect should have much higher catalytic dye decolorization efficiency than that achieved via the photocatalytic effect induced by photoelectricity. Therefore, pyroelectric materials have the potential to decolorize these organic dyes in wastewater under the alternating cold–hot temperature variation between day and night. The current reports on these pyro-catalytic materials are mainly focusing on these transitional perovskite inorganic materials, such as pyroelectric BiFeO_3_ [12], (K_0.5_Na_0.5_)NbO_3_ [13], and Bi_0.5_Na_0.5_TiO_3_ [11], etc. Room-temperature pyro-catalysis stands a good chance of being promising technique for environmental remediation. To date, there have been few reports on these organic pyro-catalyst materials.

The organic graphic carbon nitride (g-C_3_N_4_), as an analogue of graphite, has attracted considerable interest in the field of material science because of its suitable band alignment for photocatalytic water splitting, selective oxidation reactions and environmental pollutant degradation [14,15,16]. Notably, there have been no reports on its pyro-catalytic effects. Recently, two-dimensional materials, such as graphene and chalcogenide (MoS_2,_ WS_2_, etc.) nanosheets, have attracted extensive attention as high-performance piezo-/ferroelectric materials [17,18,19]. In general, the centrosymmetric crystal structure of carbon nitrides manifestly generates non-piezo-/ferroelectricity [20]. Nevertheless, the nanosheets exhibit anomalous piezo-/ferroelectricity, allowing piezo-/ferroelectricity through an interaction between symmetry and nanoscale size effects [21,22]. Meanwhile, the material performance is rooted in the energy cost of internal electric polarizations in the presence of strain gradients [21,22]. Generally, the ferroelectric material often exhibits a prominent pyroelectric performance [23,24,25,26]. Therefore, the exfoliated two-dimensional (2D) g-C_3_N_4_ organic nanosheets possess the pyroelectric effect due to the broken centrosymmetric crystal structure in theory, though the 3D bulk C_3_N_4_ are non-pyroelectric due to the limit from the centrosymmetric crystal structure.

In this study, a remarkable pyro-catalysis performance of the 2D organic g-C_3_N_4_ for Rhodamine B (RhB) dye was achieved under the room-temperature cold–hot thermal cycling from 25 °C to 60 °C, which shows potential for developing an efficient environmental remediation and wastewater disposal technology, through taking the energy of geothermal sources, industrial waste heat and the concentrated solar power to provide thermal energy [25].

## 2. Experimental Section

Materials. The reagents used were analytical grade or reagent grade without further purification prior to sample preparation. All the materials were commercially obtained (Sinopharm Chemical Reagent Co., Ltd. Shanghai, China). Deionized water was used throughout all of the experiments.

Preparation of 2D g-C_3_N_4_ organic nanosheet pyro-catalysts. The g-C_3_N_4_ nanosheets were prepared using the previous method, with minor modifications [27,28]. Firstly, bulk g-C_3_N_4_ was prepared by heating a certain amount of C_3_N_3_(NH_2_)_3_ at 550 °C in a muffle furnace for 4 h with a ramping rate of 10 °C/min [16,29]. The obtained light yellow solid product was bulk g-C_3_N_4_, which ground the natural cooling outcomes in a mortar for 20 min. Secondly, the bulk g-C_3_N_4_ was ground well and then put into HNO_3_ to be chemically oxidized. The mixed solution was sonicated and refluxed. After cooling to room temperature, the refluxed product was centrifuged and then neutralized by repeated rinsing. Thirdly, the as-obtained sediment was dispersed in the deionized water and then sonicated. After that, the suspension was centrifuged to remove the residual bulk g-C_3_N_4_. Finally, the exfoliated 2D g-C_3_N_4_ nanosheet was obtained.

Characterization. The phase structure of the synthesized g-C_3_N_4_ nanosheet catalyst was studied through powder X-ray diffraction (XRD, Philips PW3040/60, Royal Dutch Philips Electronics Ltd., Eindhoven, The Netherlands) with a Cu Kα radiation (*λ* = 1.54178 Å). The acceleration voltage was set as 40 kV and the applied current was 80 mA. The morphology of the synthesized 2D organic g-C_3_N_4_ nanosheet catalyst was observed using Hitachi H-7650 transmission electron microscopy (TEM, JEM-2100 F, Japan Electron Optics Laboratory Co., Ltd., Akishima-shi, Japan). The piezoelectric performance was characterized with piezoresponse force microscopy (PFM, Asylum Research MFP-3D, Oxford Instruments, Santa Barbara, CA, USA).

**Figure 1:**
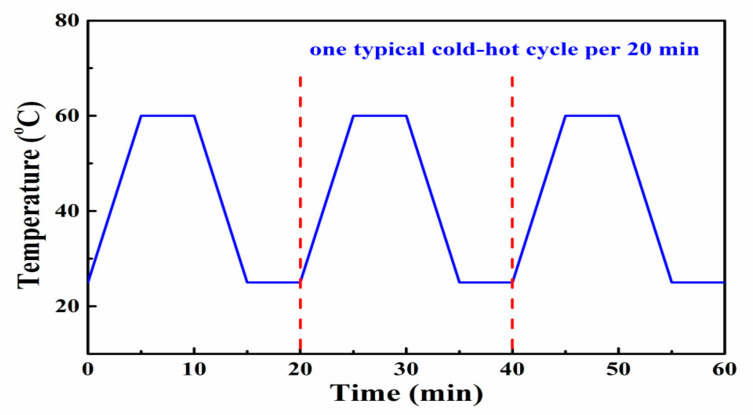
Temperature variation curve of room-temperature cold–hot cycle between 25–60 °C.

Pyro-catalytic Active Test. The decoloration of RhB dye was carried out to investigate the pyro-catalytic activities of the synthesized 2D organic g-C_3_N_4_ nanosheet catalyst. In a typical treatment, a certain amount of the 2D g-C_3_N_4_ nanosheet catalyst was suspended in a glass vessel containing 50 mL RhB dye solution with an initial concentration of 5 mg·L^−1^. All the pyroelectric catalytic decoloration tests in this work were conducted after full adsorption of RhB on g-C_3_N_4_. Prior to typical pyro-catalytic activity, the suspension was stirred in the dark for 12 h to establish an adsorption–desorption equilibrium between the 2D organic g-C_3_N_4_ nanosheet catalyst and RhB dye molecules. Therefore, the physical adsorption effect can be excluded. The solution underwent the same thermal cycles between 25 °C to 60 °C drawn in Figure 1. The cold–hot changing curve in Figure 1 is an ideal temperature controlling curve, unlike the continuous and smooth curve of the real temperature cycling curve. The sample was suspended in the solution under magnetic stirring, then the flask was applied at alternating temperatures using a water bath. In order to heat and disperse the samples, a magnetic stirring device with a heating function (RCT-B-S25, IKA, IKA Works GmbH & Co. Staufen, Germany) was applied to the reaction system. For the cooling process, a cold-water bath was used. Room temperature is a broad concept. Environmental cold–hot temperature alternation such as day–night temperatures could be classified as the scope of room temperature. The selected 25–60 °C temperature change considers our living environment temperature fluctuation. In our city, the maximum ground temperature in summer can reach 70 °C. In future, we hope to realize pyro-catalytic dye decoloration during environment temperature fluctuations, such as diurnal temperature change. Broadly speaking, we call this “room-temperature” pyro-catalysis.

For the pyro-catalytic dye decoloration studies, the concentration of RhB dye solutions was recorded, which could be identified by the initial concentrations of pyro-catalysis. The thermal treatment of the suspension was carried out. At 6 cold–hot thermal cycle intervals, 2 mL of the reaction solution was taken out and centrifuged immediately at 8000 rpm for 3 min. Then, the centrifuged solution was filtered to remove the suspended catalyst agglomerates. The concentrations of RhB dye solutions were recorded by a spectrophotometer (UV-Vis Spectrophotometer, Hitachi U-3900). The experiments were performed in the dark in order to avoid any photocatalytic RhB dye decoloration of the 2D organic g-C_3_N_4_ nanosheet catalyst material due to its visible-light response ability (the band-gap of 2D g-C_3_N_4_ is ~2.72 eV). Blank experiments were also employed in darkness following the same procedures.

Detection of superoxide radicals (•O_2_^−^) and •OH in the process of pyro-catalysis. In order to study the chemical reaction of •O_2_^−^ in the pyro-catalytic process, 1 mM of p-benzoquinone (BQ, quenching agent of •O_2_^−^) was added to design •O_2_^−^ trapping experiment [30,31]. The pyro-catalytic decolorization of RhB dye was repeated. The concentration of RhB dye was determined by a Hitachi U-3900 UV-visible spectrophotometer. The formation of the active species of •O_2_^−^ superoxide radicals in the suspension was also analyzed.

The cold–hot thermal cycling-induced active species of •OH hydroxyl radicals were confirmed by using the photoluminescence (PL) spectra of terephthalic acid generation, which was measured on the FLS920 fluorescence spectrometer (Edinburgh, UK) with an excitation wavelength of 315 nm.

## 3. Results and Discussion

**Figure 2:**
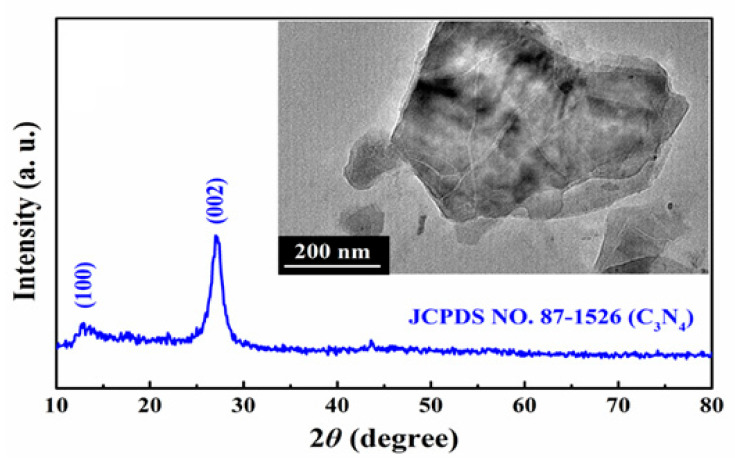
The XRD pattern of the 2D g-C_3_N_4_ organic nanosheet catalysts. The inset is the TEM picture.

The spectra (10.0–80.0 °C) XRD pattern of 2D organic g-C_3_N_4_ nanosheet catalyst powder materials are shown to identify the crystal phase structures of the as-prepared 2D organic g-C_3_N_4_ nanosheet in Figure 2a. These distinct diffraction peaks at 13.0° and 27.4° indicate stacking of aromatic system in the upper half of Figure 2, being indexed as the (100) and (002) diffraction crystal planes to C_3_N_4_ (JCPDS Card No. 87-1526) [32]. No impurity peaks are detected, indicating that the synthesized 2D organic g-C_3_N_4_ nanosheet catalyst powder material products contain pure phase.

Furthermore, the morphologies and sizes of the 2D organic g-C_3_N_4_ nanosheets characterized by TEM are demonstrated in the picture embedded in Figure 2. The synthesized 2D g-C_3_N_4_ organic catalyst nanomaterials are sheet particles, while the layer morphology of the 2D organic g-C_3_N_4_ nanosheet catalyst is the key to affecting the pyroelectric performance [17,22]. It can be seen from the TEM result in the inset of Figure 2 that the 2D organic g-C_3_N_4_ nanosheet catalyst is very thin. This is conducive to more exposure of reactive sites, which is beneficial to the separation and transport of these pyroelectrically induced positive and negative electric charges, improving the dye decolorization efficiency of the pyro-catalytic reaction.

**Figure 3:**
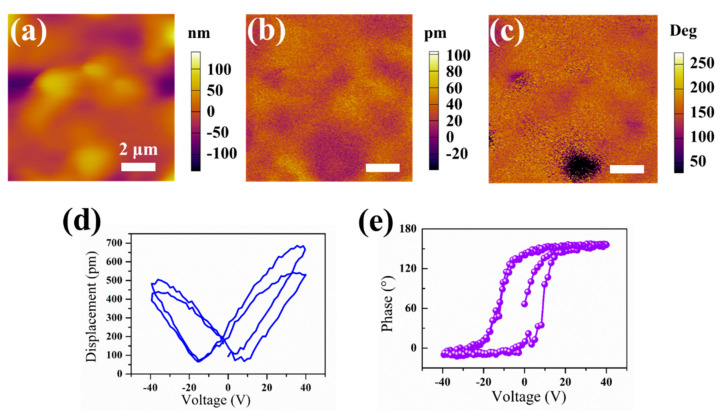
(**a**) Topography, (**b**) PFM amplitude, (**c**) PFM phase images of the 2D organic g-C_3_N_4_ nanosheet catalyst. (**d**,**e**) the local piezoelectric hysteresis loops of the 2D organic g-C_3_N_4_ nanosheet catalyst.

Ferroelectricity is applicable to materials with spontaneous electrical polarization that can be reversed by the application of an external suitable electric field. The neat electric dipolar alignment can be broken under the external stress or thermal excitation, which can change the polarization strength and break the charge balance on the ferroelectric material’s surface, resulting in the occurrence of the piezoelectric effect or the pyroelectric effect. Therefore, all ferroelectric materials are pyroelectric and all pyroelectric materials are piezoelectric [29,30,31,32]. The ferroelectric/piezoelectric response of the 2D organic g-C_3_N_4_ nanosheet catalyst was characterized through the PFM. The topography shows the 2D organic g-C_3_N_4_ nanosheet catalyst has a flat and smooth surface (Figure 3a). The corresponding PFM amplitude and phase images show a clear contrast, which suggests that the 2D organic g-C_3_N_4_ presents ferroelectric domains as shown in Figure 3b,c. Well-defined butterfly loops of the PFM amplitude signals corroborate the good piezoelectric response of the 2D organic g-C_3_N_4_ nanosheet catalyst materials (Figure 3d). The phase angle of the g-C_3_N_4_ shows obvious and typical ferroelectric hysteresis and a 170° change under direct current bias field voltages ranging from −40 V to +40 V, revealing the ferroelectric polarization switching process of the 2D organic g-C_3_N_4_ nanosheet catalysts (Figure 3e). Thus, the pyroelectric performance of the 2D organic g-C_3_N_4_ nanosheet catalyst materials can be expected.

**Figure 4:**
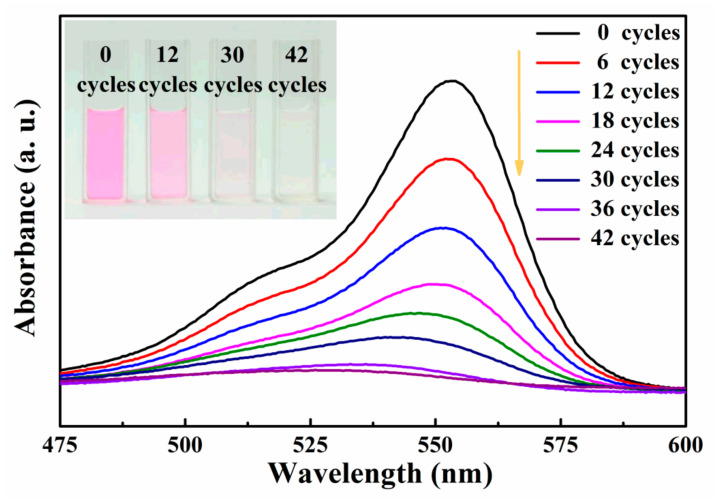
The absorbance spectra of RhB dye solution with pyro-catalysis of the 2D organic g-C_3_N_4_ nanosheet catalyst material. The inset is the picture of color change of RhB dye solution.

To test g-C_3_N_4_ nanosheets’ ability to generate electric potential under temperature changes, a pyro-catalytic experiment was conducted to degrade RhB dye. The experiment involved exposing the g-C_3_N_4_ nanosheets and the RhB dye solution to a cold–hot alternation cycle and measuring the decolorization rate of the dye. The color change indicates the degree of decolorization and the removal of organic pollutants from the dye solution. By observing the color change of RhB dye, the effectiveness of the organic 2D g-C_3_N_4_ nanosheets in catalyzing the degradation of the dye under temperature changes can be easily perceived. Figure 4 displays the changes in the absorption spectra of RhB after being subjected to various numbers of cold–hot thermal cycles in the presence of the 2D organic g-C_3_N_4_ nanosheet catalyst. It can be seen that the characteristic absorption spectrum of RhB dye is about 554 nm, corresponding to the π-π* transition of the conjugated system in RhB molecules [33]. As the number of cold–hot thermal cycles increase, the absorption intensity of RhB dye solution decreases sharply. The absorption spectra reflect the concentration of RhB molecules in the solution. The decrease in the absorption peaks indicates the decolorization of RhB by the pyro-catalytic action of g-C_3_N_4_ nanosheets. As shown in the inset of Figure 4, we can visually see the decolorization progress through the color change of the RHB solution during pyro-catalysis. Apparently, after 42 cold–hot thermal cycles, the color of RhB dye solution gradually fades from the initial pink to colorless.

The decolorization efficiency (*D*) of RhB dye can be calculated by the ratio of the absorbance value over time to the initial absorbance, as shown in Equation (1) below,

*D* = (*C*_0_ − *C*)/*C*_0_ × 100% (1)

where *C*_0_ represents the initial concentration of RhB dye solution. *C* is the concentration of RhB dye solution after experiencing different cold–hot thermal cycles. From Equation (1), the pyro-catalytic decoloration efficiency of RhB dye in the presence of the 2D organic g-C_3_N_4_ nanosheet catalyst is calculated as high as ~92.6% for 42 cold–hot thermal cycle times. When discussing the pyro-catalysis performance, it should be noted that it can be deeply affected by many factors such as the temperature changing velocity, the cold–hot alternation temperature range, the catalyst content, the initial dye solution’s concentration and so on [11,12,13].

**Figure 5:**
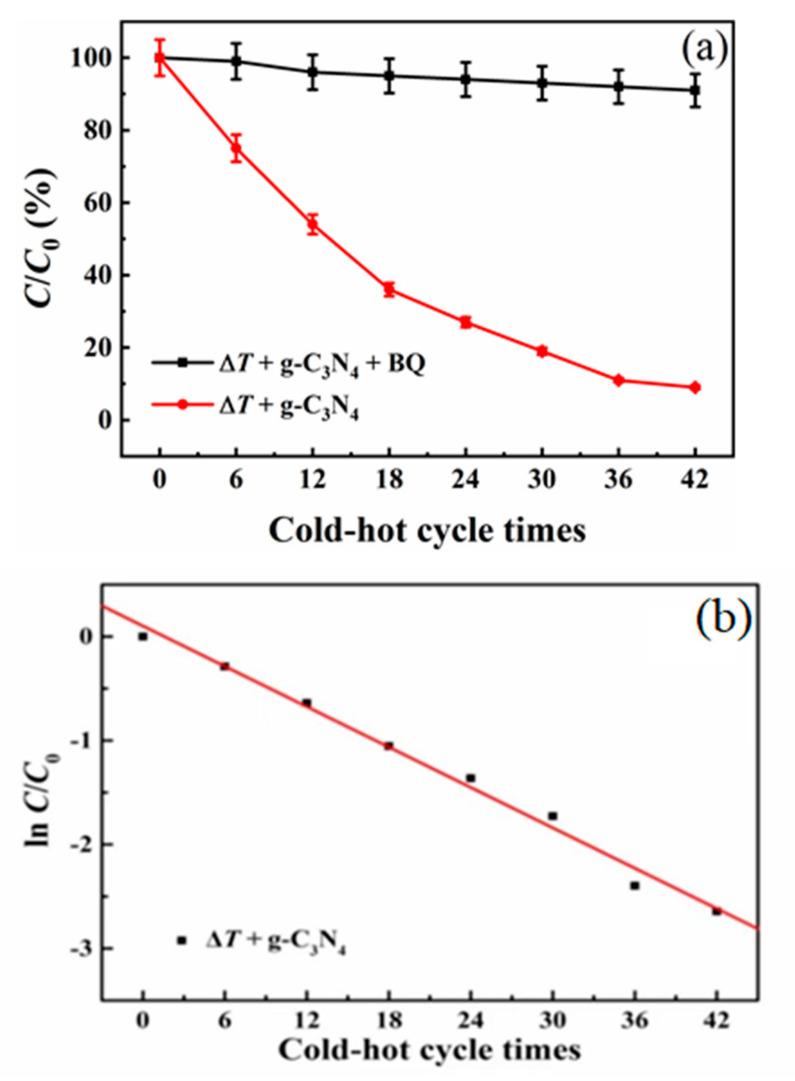
(**a**) The decoloration efficiency of RhB dye. (**b**) The curve of ln(*C*_0_/*C*) vs. cold–hot cycle times.

As shown in Figure 5a, the specific value *C*/*C*_0_ vs. cold–hot cycle times in the presence of g-C_3_N_4_ has been plotted, which shows an exponential behavior with cold–hot cycle times. An attempt is made to fit the data using some common kinetic equations with the purpose of determining the reaction rate. The pseudo first-order kinetics of RhB dye decoloration in the presence of 2D organic g-C_3_N_4_ nanosheets is confirmed by making a linear plot of ln(*C*_0_/*C*) vs. the cold–hot thermal cycle times, as shown in Figure 5b. The kinetic equation can be expressed as the following Equation (2) [34,35],

ln(*C*/*C*_0_) = −*k*·*t* (2)

where *t* and *k* represent different cold–hot cycle times and the rate constant determined from the minus slope of ln(*C*_0_/*C*) vs. the cold–hot cycle times, respectively. The rate constant of g-C_3_N_4_ is determined from Equation (2) to be ~6.5 × 10^−2^ time^−1^, confirming the pseudo first-order kinetic characterization of RhB decoloration in the presence of 2D organic g-C_3_N_4_ nanosheet catalyst.

In order to study the chemical reaction of •O_2_^−^, BQ was used as •O_2_^−^ trapping agent in the pyro-catalytic RhB decoloration in the presence of the organic 2D g-C_3_N_4_ nanosheet catalysts. The pyro-catalytic decolorization of RhB dye was repeated [30,31]. The decoloration efficiency curve *C*/*C*_0_ vs. the cold–hot cycle times of RhB dye can be seen in Figure 5a. The addition of BQ (the quencher of •O_2_^−^) mediated by the 2D organic g-C_3_N_4_ pyro-catalyst material significantly inhibits the decoloration of RhB dye, indicating that active •O_2_^−^ is the primary reactive species in the pyro-catalysis process [36,37].

**Figure 6:**
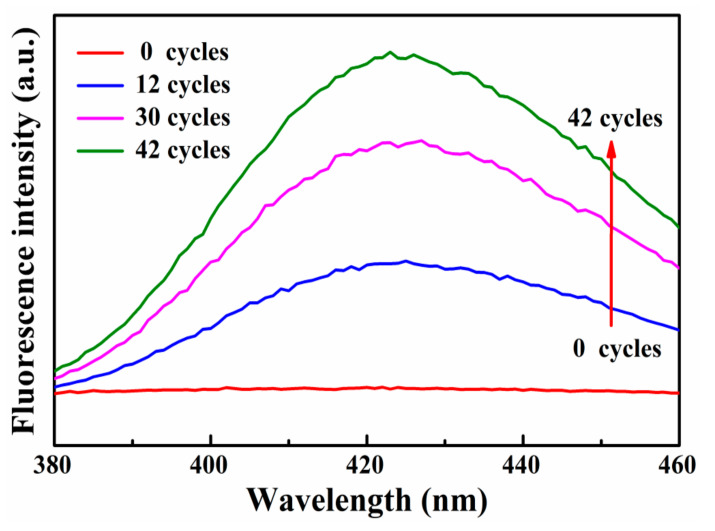
The fluorescent signals of 2-hydroxyterephthalic acid.

Furthermore, •OH was also observed as middle products of pyro-catalytic RhB dye decoloration in the presence of the 2D organic g-C_3_N_4_ nanosheet catalysts. In the experiment, the terephthalic acid was used as the photoluminescent •OH trapping agent. The •OH reacts with the terephthalic acid to form a highly fluorescent product, 2-hydroxyterephthalic acid, which has a characteristic fluorescence peak at the wavelength of ~425 nm [38,39]. The significant fluorescent signals associated with 2-hydroxyterephthalic acid are shown in Figure 6. The 2D organic g-C_3_N_4_ nanosheets produce positive and negative electric charges after experiencing different thermal cold–hot cycles to generate the active species of hydroxyl radicals. The hydroxyl radicals combined with terephthalic acid to produce 2-hydroxyl terephthalic acid, which shows an obvious fluorescence signal. As shown in Figure 6, the amount of •OH produced during the pyro-catalytic process is proportional to the strength of the 2-hydroxyterephthalate PL peak [38,39,40], which indicates that the •OH are one of the middle active species in the process of pyro-catalysis of the 2D organic g-C_3_N_4_ nanosheet catalysts.

In a word, •O_2_^−^ and •OH radicals are the active species that efficiently deal with the RhB solution. The whole electronic transition and pyro-catalytic dye decoloration reaction is described as follows in Equations (3)–(6),

g-C_3_N_4_ + ΔT → *q*^−^ + *q*+ (3)

O_2_ + *q*^−^ → •O_2_^−^ (4)

OH^−^ + *q*^+^ → •OH (5)

•O_2_^−^ (or •OH) + RhB→ dye decoloration (6)

During the cold–hot thermal cycling process, the pyroelectric g-C_3_N_4_ nanosheet catalysts produce some positive and negative electric charges (Equation (3)). The pyroelectrically induced positive and negative electric charges can further react with hydroxide ions and dissolved oxygen in RhB dye solution to generate •O_2_^−^ and •OH radicals, respectively (Equations (4) and (5)). •O_2_^−^ and •OH radicals strong oxidizing properties to decolorize RhB dye solution (Equation (6)).

The pyroelectric effect is mainly aroused by the temperature gradient (Δ*T*). The thermal energy (Δ*Q*) provided into the pyro-catalytic system from the surrounding environment can be calculated on the basis of Equation (7),

Δ*Q* = *c*·*m*·Δ*T* (7)

where *c* and *m* represent specific heat capacity and mass of the organic dye solution, respectively. Under the continuous cold–hot cycle excitation, the pyroelectric materials always act as the following characteristics. In thermodynamic equilibrium, compensation charge carriers screen the bound polarization charges completely [41]. With a dynamic temperature, a surface potential develops on account of a transience between electrical polarization and screened charges [42]. When the surface charges density surpasses the electrical polarization charge density, the middle products of some molecular species, such as OH^−^ and O_2_, undergo redox reactions to generate these active species’ •O_2_^−^ and •OH radicals, resulting in the decoloration of RhB dye [43].

In general, the other inorganic perovskite pyro-catalysts often possess a high density. One example of this is BaTiO_3_, which has a density of 6.08 g·mL^−1^ [11,12,13]. However, it is difficult to recycle the organic pyro-catalyst because the 2D organic g-C_3_N_4_ nanosheet catalysts are very lightweight with a density of about 0.699 g·mL^−1^. In future, the stability and recyclability of the 2D g-C_3_N_4_ nanosheet material should be further studied in detail for the practical application of the organic pyro-catalysts in dye wastewater treatment.

In future, there is hope of further enhancing the pyro-catalysis performance of the 2D organic g-C_3_N_4_ catalyst through building a heterojunction structure to increase the separation of pyroelectrically induced positive and negative charges [44,45,46,47]. Besides the pyro-catalysis for harvesting environmental cold–hot thermal energy, the 2D organic g-C_3_N_4_ nanosheet catalyst materials can be also designed to harvest mechanical energy to drive a chemical catalysis reaction [48,49]. The 2D organic g-C_3_N_4_ catalyst nanomaterials have been reported as having an excellent piezocatalytic performance [48]. In our previous report, we have also found that the 2D organic g-C_3_N_4_ could exhibit a strong tribocatalytic performance for nitrogen fixation through harvesting environmental friction energy [49]. It has the potential to obtain the enhanced catalytic dye decoloration performance of the 2D organic g-C_3_N_4_ nanosheet catalyst nanomaterials by harvesting both the environmental cold–hot temperature alternation thermal-energy and the ordinary mechanical friction energy and vibration energy on basis of the synergy of muti-catalysis in future [50,51,52,53,54].

## 4. Conclusions

In summary, an obvious pyro-catalytic performance was achieved in the 2D organic g-C_3_N_4_ nanosheet catalyst materials under a room-temperature cold–hot alternating excitation (between 25 °C to 60 °C) through a combination of the pyroelectric effect and the electrochemical redox reaction. The pyro-catalytic decoloration efficiency of the 2D organic g-C_3_N_4_ nanosheet catalyst for RhB dye can reach ~92.6% in the dark. Superoxide radicals and hydroxyl radicals as the intermediate products in the pyro-catalysis are observed and conformed, decoloring the RhB dye solution. Herein, the perception of pyro-catalysis of the 2D organic g-C_3_N_4_ nanosheet catalyst materials provides efficient and reusable technology for wastewater applications by utilizing environmental cold–hot thermal energy in nature.

## Data Availability

The data presented in this study are available on request from the corresponding authors.
